# Silver dressings for the healing of venous leg ulcer: A meta-analysis and systematic review

**DOI:** 10.1097/MD.0000000000022164

**Published:** 2020-09-11

**Authors:** Minyan Zhao, Dongting Zhang, Liping Tan, Hui Huang

**Affiliations:** a Department of Oncology, The Second Affiliated Hospital of Soochow University; b School of Nursing, Soochow University; c Department of Nursing, The Second Affiliated Hospital of Soochow University, Suzhou, China.

**Keywords:** silver dressings, venous leg ulcer, wound healing, meta-analysis

## Abstract

This study was aimed to evaluate whether silver-containing dressings were superior to other types of dressings in the treatment of venous leg ulcers (VLU) and their specific advantages.

Eight databases (Cochrane Library, PubMed, Web of Science, Ovid-Medline, Wanfang, VIP, China Biology Medicine, and China National Knowledge Infrastructure) were systematically reviewed from inception to May 2019 for randomized controlled trials (RCTs). The primary outcome was complete wound healing, and the secondary outcomes included absolute wound size changes (change of cm^2^ area since baseline), relative changes (percentage change of area relative to baseline), and healing rate. Two reviewers independently evaluated the risk of bias using the Cochrane Collaboration assessment tool and extracted the data according to the predesigned table. All analyses were performed using the latest Review Manager Software (version 5.3).

A total of 8 studies qualified and were included in the meta-analysis, including 1057 patients (experiment: 526, control: 531). Both complete wound healing and wound healing rates were reported in 5 studies. Two and 3 studies reported the effect of silver dressings on absolute and relative wound size changes, respectively. Most of the studies used intention-to-treat analysis.

There was sufficient evidence that silver-containing dressings can accelerate the healing rate of chronic VLU and improve their healing in a short duration of time. However, compared with other dressings, clinical trials with long-term follow-up data are needed to confirm whether silver dressings have advantages regarding complete wound healing.

## 1 Introduction

Venous Leg Ulcer (VLU) is a common type of chronic wound, and is always accompanied by long course, slow healing and easy recurrence.^[1]^ A chronic venous ulcer is the most severe manifestation of chronic venous insufficiency and accounts for the vast majority of lower limb ulcerations.^[2]^ The incidence of VLU increases progressively with age and is estimated to be 1% to 3%, in the adult population.^[3]^ Moreover, venous ulcer can lead to pain, activity restriction, sleep disturbances, and other problems, which can seriously affect the quality of life of patients; the high cost of treatment is also a huge economic burden on patients and society.^[4,5]^

Multilayer compression therapy is currently considered to be the gold standard for VLU treatment.^[6,7]^ Wound contact dressings are usually placed underneath the compression devices and play a key role in promoting ulcer healing.^[8,9]^ Several studies have demonstrated that in patients with VLUs, wounds may last for several years without any real improvement.^[10,11]^ It is a well-known fact that, infection is a major cause for slow wound healing and failure to heal.^[12]^ Silver, as a broad-spectrum antimicrobial agent, covers almost all bacteria that colonize chronic wounds. In addition, silver ion has a strong anti-inflammatory effect, and could also inhibit the metalloproteinases activity and promotes senescent cells apoptosis. Resistance to the silver ion rarely occurs due to its complex mechanism of action.^[13]^ Therefore, silver-containing dressings have become increasingly popular for wound care in clinical practice.^[14]^

Silver has a long history for wound management, but scientific evidence of its efficacy is lacking. A systematic review published in Cochrane Library in 2010 showed that there was insufficient evidence to determine whether silver dressings could promote wound healing or prevent wound infection.^[15]^ However, Marissa et al showed that there is strong evidence that silver-containing dressings or local silver agents can facilitate wound area reduction.^[16]^ Furthermore, a meta-analysis published in 2017 including 31 randomized controlled trials (RCTs) and 8 cohort studies pointed out that the role of silver in wound treatment is significantly better than what was recognized in current scientific debates. If used correctly, silver not only has antimicrobial effects, but is also cost-effective and can improve the quality of life of the patient.^[17]^ It is evident that the effect of silver in wound care has always been controversial and the effect of silver in patients with venous ulcer was not fully understood. Therefore, the purpose of this meta-analysis was to evaluate whether silver-containing dressing is superior to other types of dressings in the treatment of VLU, and also to elucidate its specific advantages.

## 2 Methods

### 2.1 Search strategy

Ethical review is not applicable for the current study, since all the data analyzed in this study acquired from published papers. This meta-analysis was performed based on the Preferred Reporting Items for Systematic Reviews and Meta-Analysis (PRISMA).^[18]^ RCTs published from inception of the databases to May 2019 were retrieved. The Cochrane Library, PubMed, Web of Science, Ovid-Medline, Wanfang, VIP, China Biology Medicine (CBM), and China National Knowledge Infrastructure (CNKI) databases were systematically searched without any language limitations. The following search terms were used: “silver dressing” or “silver-based” or “silver-releasing” or “silver-impregnated” or “silver-containing” or “silver-donating” or “silver” in combination with “venous ulcer” or “leg ulcer” or “varicose ulcer” or “crural ulcer” or “stasis ulcer” or “VLU” Two reviewers performed a preliminary screening of the studies by reading the titles and abstracts. Full texts of articles that seemed to meet the inclusion criteria were obtained for further assessment. Additionally, the references of included studies were also searched.

### 2.2 Participants

Patients diagnosed with venous ulcer, without location or grade limitation were included. Studies were also included if the data of patients with venous ulcer could be extracted separately, or a predominant (≥70%) proportion of the participants in both groups (cases and controls) had leg ulcers of venous etiology.

### 2.3 Interventions

The experimental group was treated with various types of dressings containing silver, whereas the control group was treated with other types of dressings or local preparations. Both groups should have been treated with pressure therapy.

### 2.4 Outcomes

The primary outcome was that the ulcers were completely healed. The secondary outcomes included absolute wound size changes (change in cm^2^ area since baseline), relative changes (percentage change in area relative to baseline), healing rate (e.g., cm^2^/week), and infection rate or reduction in infection. At least one of these outcomes should have been included in the trial.

### 2.5 Wound dressings

The classification of dressings usually depends on the key material in their construction. In the current study, all dressings containing silver were classified as the experimental group regardless of other characteristics. Usually, in the control group, the dressing had similar characteristics with the test dressing and the only difference was the silver content. The control group was divided into 3 subgroups according to the dressing characteristics: the traditional dressing group, the antibacterial dressing group and the other modern dressing group. The traditional dressing for the treatment of venous ulcer mainly refers to Vaseline gauze. It would not adhere to the wound, but can not promote the whole healing process of the wound. Antimicrobial dressings are composed of a gauze or low-adherent dressing impregnated with an ointment thought to have antimicrobial properties.^[19]^ They are mostly used in chronic wounds and control wound infection. Modern dressings involve a series of dressings with special functions, including foam dressing, hydrocolloid dressing, alginate dressings and so on. Their functions include, but are not limited to, absorbing and containing exudates, optimizing wound pH, and relieving pain.

### 2.6 Data extraction

Two reviewers independently extracted the data according to the pre-designed table, which included the general characteristics of studies, key baseline participant data (age, gender, ulcer size, ulcer duration), number of participants, details of dressings or local preparations, duration of trials, primary and secondary outcomes, and withdrawal numbers.

### 2.7 Quality assessment

Two reviewers independently evaluated the risk of bias of included RCTs using the assessment tool provided by Cochrane Collaboration,^[20]^ which assesses the following parameters: “selection bias, performance bias, detection bias, attrition bias, reporting bias, and other biases”. Each aspect was evaluated in terms of “high-risk,” “low-risk,” and “unclear.” Disagreements were discussed between the 2 reviewers and a third reviewer provided assistance in judging to reach a consensus, if necessary.

### 2.8 Statistical analysis

Meta-analysis was performed using the latest Review Manager Software (version 5.3). We used the risk difference (RD) and 95% confidence interval (CI) to calculate the results of dichotomous variables. Continuous variables were determined by weighted mean difference (WMD) or standardized mean difference (SMD) and their 95% CI. Chi-Squared test (Q test) was used to judge the heterogeneity of these studies. If *P* > .1 and *I*^2^ < 50%, data were considered homogenous and fixed-effects model was adopted. If *P* < 0.1 and I^2^ ≥ 50%, the random-effects model was adopted. If *P* < .1 and the source of heterogeneity could not be determined, or the outcomes could not be combined due to the inconsistent presentation methods, only descriptive analysis was performed on the data. Sensitivity analysis was used to investigate the effects of fixed-effects or random-effects models on heterogeneity. If sufficient number of studies were included, a funnel plot would be used to investigate publication bias.

## 3 Results

### 3.1 Literature search

**Figure 1:**
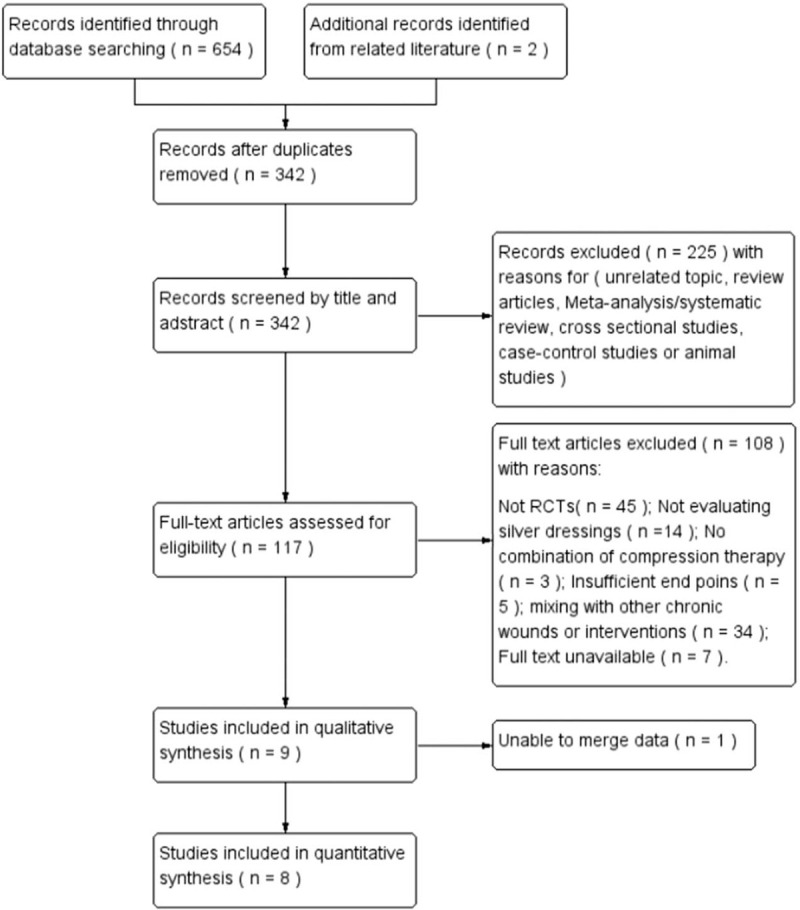
Flow chart of search results.

A total of 654 relevant studies were obtained by preliminary search of the literature and 2 related studies were supplemented by reading previous articles. After eliminating the duplicates, 342 papers remained. Then, 225 articles were excluded after reading the titles and abstracts due to apparent non-compliance with inclusion criteria. Finally, 117 articles were selected for full-text review, of which 108 articles were excluded for the following reasons: non-randomized studies (n = 45), not evaluating silver dressings (n = 14), not in combination with pressure therapy (n = 3), mixing with other chronic wounds or interventions (n = 34), insufficient end points (n = 5), and full text unavailable (n = 7). Therefore, 9 RCTs were included in qualitative synthesis, of which data in 1 RCT was unable to be integrated. Finally, 8 RCTs were included in this study^[8,13,21,22,24–27]^ (Fig. 1).

### 3.2 Characteristics of the included trials

A total of 1057 participants among the 8 RCTs were included in this study. The RCTs were conducted in France (n = 2),^[13,21]^ Britain (n = 1),^[8]^ Poland (n = 1),^[22]^ Australia (n = 1),^[23]^ China (n = 2),^[25,26]^ and 1^[24]^ was a multinational trial in 5 Western countries. Details of the baseline characteristics of each study are provided in Table 1. The average age of participants ranged from 60 through 80, except for 1 study in which age of participants ranged from 18 through 90. The baseline size of ulcer varied from 6 cm^2^ to 47 cm^2^, and ulcer duration also varied from a month to about 3 years. In the RCT of Kerihuel et al,^[21]^ the ankle brachial pressure index (ABPI) of patients was above 0.7. Twenty seven (45%) of patients were already being treated with compression at inclusion and 22 (36.7%) had edema. Sixteen (53.3%) and 19 (63.3%) patients given the test and control dressing respectively had a history of ulceration. The research conducted by Krasowski et al^[22]^ required the ABPI of participants above 0.8 and the leg wounds, which were 2 to 200 cm^2^, did not heal for at least 6 weeks. The requirements of Lazareth et al^[13]^ for ABPI of participants were consistent with Krasawski et al. In their study, leg ulcers were present for almost 11 months on average (median 9.0 months) and 65% were recurrent. 51 ± 28% of the wound surfaces were covered with sloughy tissue (yellow appearance on colourimetric scale) and 2.9% presented with healthy perilesional skin. In the research carried out by Michaels et al,^[8]^ overall 28.2% of the ulcer size was more than 3 cm and 38.0% of ulcers lasted longer than 12 weeks. A total of 53.1% patients reported previous episodes of leg ulceration. In a relatively big RCT by Miller et al,^[23]^ the ABPI of patients was above 0.6 and the wound was 15 cm or less in diameter. In addition, patients had at least one signs of infection or critical colonization of the wound. In the research of Senet et al,^[24]^ the ABPI of participants was above 0.8 and ulcers were between 2 cm and 13 cm in all directions or the ulcers have been properly treated within 4 weeks before recruitment, but the ulcer size reduced less than 20%. Zhang et al^[25]^ reported the difference of baseline data between the 2 groups was not statistically significant (*P* > .05). However, the study did not show us any information about the wounds at baseline. Zhou et al^[26]^ chose the patients with venous leg ulcer VLU who first went to the outpatient treatment center of the hospital. The ulcer lasted for 1 to 3 months. And the average wound area was 46.58 ± 0.68 cm^2^ in the observation group and 47.13 ± 0.43 cm^2^ in the control group.

**Table 1:**
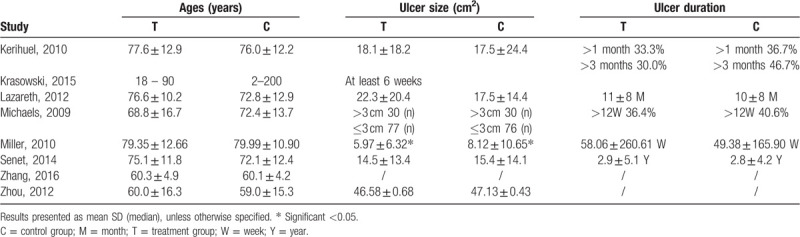
Baseline characteristics: participants and ulcer wounds.

All studies reported the role of silver dressings in VLU wound healing. Five studies^[8,23–26]^ compared effects of silver-containing dressings on complete wound healing. Two^[13,21]^ and 3^[13,21,24]^ studies reported the effect on absolute and relative wound size changes, respectively. Five studies^[13,22–24,26]^ analyzed the effect on wound healing rate. Four studies had a sample size of 60^[21,26]^ or 80,^[22,25]^ while the other 4 studies had participants ranging from 102 through 281.^[8,13,24,25]^ The duration of each trial ranged from 3^[26]^ through 12^[8,23]^ weeks and the drop out rate ranged from 0^[25,26]^ through 16.7%^[13]^ (Table 2).

**Table 2:**
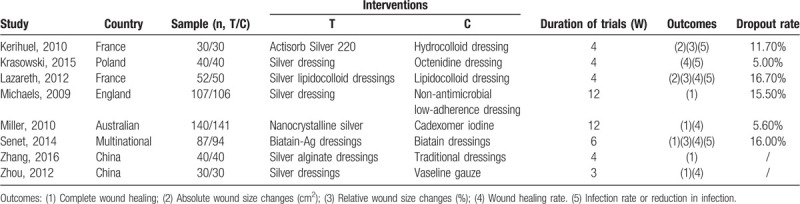
Details of studies included in the systematic review.

### 3.3 Wound dressings

In the study of Kerihuel et al,^[21]^ the hydrocolloid dressings were used in the control group, which were usually a breathable membrane or foam pad made of water absorbent colloidal matrix. While in the study of Krasowski et al^[22]^ and Miller et al,^[23]^ antibacterial dressings were used as control. The main antibacterial substances were iodine and iodine respectively. The lipidodoloid dressings used in Lazareths research^[13]^ were composed of a polyester textile mesh impregnated with hydrocolloid particles and Vaseline, and the non-silver low-adherence dressings used in Michaelss study^[8]^ usually consist of cotton pads that are placed directly in contact with the wound. The study of Senet et al.^[24]^ used Biatain dressings, which are made of hydrophilic polyurethane hydrocellular and are covered by a plain polyurethane Biatain topfilm. In addition, 2 studies^[25,26]^ used traditional dressings as control. Due to the small number of studies included, this meta-analysis would not be grouped according to the dressing characteristics of the control group.

### 3.4 Risk of bias

**Figure 2:**
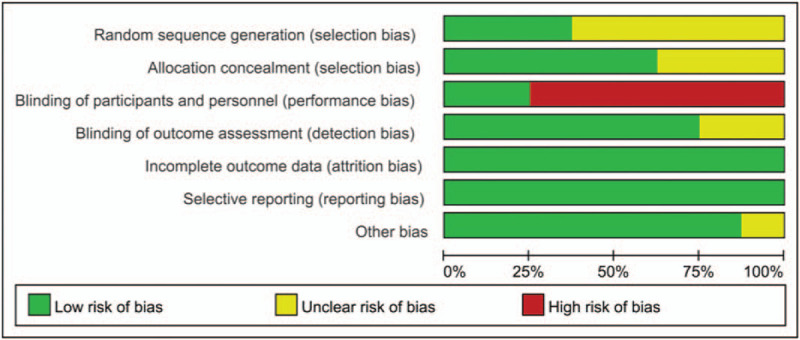
Overall risk of bias assessment using the Cochrane tool.

**Figure 3:**
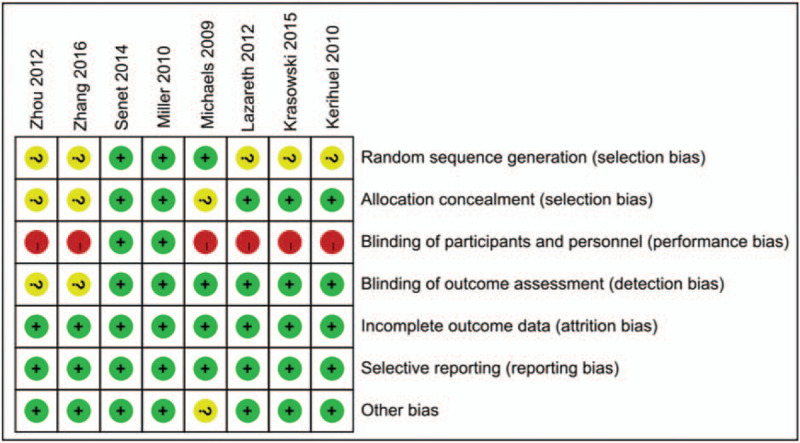
Risk of bias assessment by individual trials.

The risk of bias across the 8 included RCTs is shown in Figures 2 and 3. All studies had a low risk of bias regarding incomplete outcome data and selective reporting. Three studies^[8,24,25]^ which reported the random sequence generation in detail had a low risk of bias. The risk of bias in the remaining 5 studies was unclear. As for the allocation concealment and the blinding of outcome assessment, 5^[13,21,22–24]^ and 6^[8,13,21–24]^ studies had a low risk of bias, respectively. Only 2 studies^[23,24]^ mentioned the blinding of participants and personnel and 6 other studies, were considered to have a high risk, although they did not mention this point. In terms of other biases, 7^[13,21–26]^ studies had a low risk. Because only 8 articles were included in this study, no funnel plot analysis was conducted, so it was not possible to determine whether there was potential publication bias.

### 3.5 Analysis of complete wound healing

**Figure 4:**
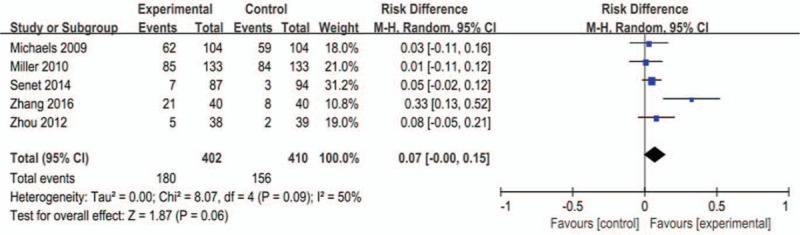
Effects of silver-containing dressings on complete wound healing.

Five studies^[8,23–25]^ reported complete wound healing. Statistical heterogeneity was present among the studies (*P* = .09, *I*^2^ = 50%), so the random-effects model was used. Meta-analysis demonstrated that silver dressings had no meaningful effect on the proportion of ulcers completely healed, and there was no statistical significance in the combined effect (RD = 0.07, 95% CI [–0.00, 0.15], *P* = .06, Fig. 4).

### 3.6 Analysis of absolute wound size changes

Two studies^[13,21]^ reported an absolute reduction in ulcer size. However, due to the differing presentation of the outcome, only descriptive analysis was carried out. In a study by Kerihuel et al,^[21]^ the median area of ulcer reduction in the silver dressing group was – 4.5 (– 30.9, – 22.5) cm ^2^ at the fourth week, which was higher than that in the control group – 3.5 (– 53.3, – 18.5) cm ^2^. Lazareth et al^[13]^ showed that the ulcer area of the experimental group decreased (6.5 ± 13.4 cm^2^) at the fourth week, which was higher than that of the control group (1.3 ± 9.0 cm^2^). The difference was statistically significant (*P* = .023).

### 3.7 Analysis of relative wound size changes (percentage)

**Figure 5:**
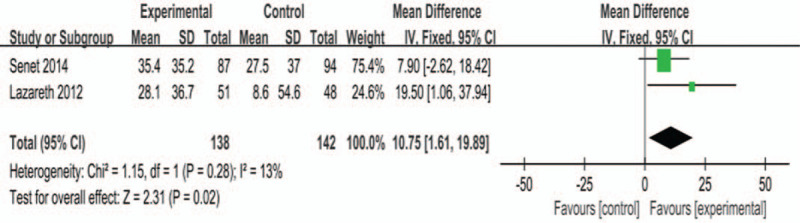
Effects of silver-containing dressings on relative wound size changes.

Three studies^[13,21,24]^ reported relative reductions in ulcer size. One^[21]^ of the studies differed in the presentation of the outcome, so we did a descriptive analysis for this study. There was no statistical heterogeneity (*P* = .28, *I*^2^ = 13%) between the 2 RCTs entered in the meta-analysis, so the fixed-effects model was used. Meta-analysis showed that silver dressing could improve the relative reduction in ulcer size, and the combined effect was statistically significant (MD = 10.75, 95% CI [1.61, 19.89], *P* = .02, Fig. 5). When the same data were reanalyzed using a random-effects model, the results were still statistically significant (MD = 11.13, 95% CI [0.94, 21.31], *P* = .03). In the study by Kerihuel et al, the median ulcer area reduction rate of the silver dressing group was – 35.6 (– 100, – 182.1)% at the fourth week, lower than that of the control group, which was – 40.9 (– 100, – 308.3)%.

### 3.8 Analysis of healing rate

**Figure 6:**
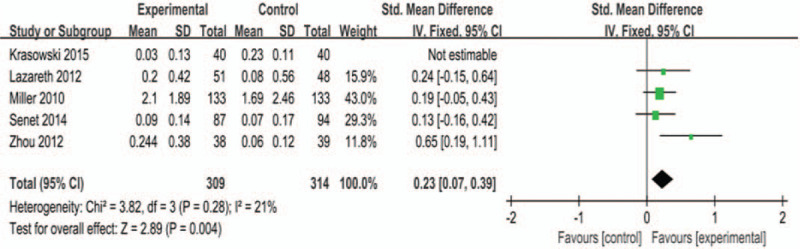
Effects of silver-containing dressings on healing rate.

Five studies^[13,22,23–26]^ reported healing rate (per day) of ulcers. There was significant heterogeneity among the studies (*P* < .01, *I*^2^ = 92%). After analysis, we found that the source of heterogeneity may have been related to the dressings used in the control group. Among the 5 studies included, 1 study^[22]^ used octenidine dressing with strong antimicrobial ability in the control group, while the other 4 studies used dressings without antimicrobial activity or with general antimicrobial activity. Therefore, these 4 studies were analyzed by meta-analysis, and the 1 with use of octenidine was analyzed by descriptive analysis. There was no statistical heterogeneity (*P* = .28, *I*^2^ = 21%) between the 4 RCTs entered in the meta-analysis, and the fixed-effects model was used. Meta-analysis suggested that silver dressings could improve the healing rate of ulcers, and the combined effect was statistically significant (MD = 0.23, 95% CI [0.07, 0.39], *P* = .004, Fig. 6). When the same data were reanalyzed using a random-effects model, the results were still statistically significant (MD = 0.24, 95% CI [0.06, 0.43], *P* = .009).

### 3.9 Analysis of infection rate or reduction in infection

Four studies^[13,21,22,24]^ reported the information about wound infection. However, due to the differing presentation of the outcome, only descriptive analysis was carried out. In the study by Kerihuel et al,^[21]^ there was 1 wound infection in both the silver dressing group and the control group. Krasowski et al^[22]^ showed that on the 28th day of the trial, microbiological eradication was observed among 6% (1/32) of patients in the silver dressing group vs 33%(5/23) in otinidine dressing group. The difference was not statistically significant (*P* = .08). Lazareth et al^[13]^ indicated no infection occurred in the silver group treatment vs 1 infection in the control group within 4 weeks. Senet et al^[24]^ reported that the frequency of patients reporting at least 3 out of 5 pre-defined local inflammatory signs (pain, odour, erythema, oedema, and exudate) were equal in both the groups after 6 weeks treatment.

## 4 Discussion

Chronic venous ulcer of lower extremity is a common chronic disease prone to recurrence, accompanied by varying degrees of chronic pain, which seriously affects sleep and quality of life of patients. Though the application of silver dressings in the treatment of VLU has gradually become popular, and has progressively increased in recent years, the specific effect of this dressing on wound healing is still uncertain or controversial.^[27]^ This may explain why this study only focuses on wound healing parameters.

Overall, the quality of the 8 RCTs included in this study was relatively good. Most of the studies used intention-to-treat analysis, and explained the detailed reasons for each persons withdrawal. Though only 2 studies^[23,24]^ explicitly mentioned the use of double blindedness, most^[8,13,21–24]^ of the outcomes were measured using blind methods. However, most studies had the problem of short intervention time. Though several studies^[13,22,24]^ were conducted for a relatively long time (8–10 weeks), silver dressings were used only in the first 4 weeks, making long-term follow-up data unavailable. Finally, we found that different RCTs had different or even contrasting results related to the same outcome, which made it almost impossible to obtain a strong recommendation without meta-analysis.

In this meta-analysis, there was no significant difference in complete wound healing between the experimental group and control group (*P* = .06). This may have been related to the duration of intervention. Because of the high cost of silver dressings and the difficulty of long-term follow-up, RCTs for evaluating silver dressings usually lasted for about several weeks, rather than a couple of months, which is usually the time needed for chronic wound healing.^[16]^ In the current research, 5 original studies^[8,23–26]^ reported the proportion of complete healing of ulcer wounds, 3^[24–26]^ of which were treated with silver dressings within 6 weeks. Therefore, we believe that in order to observe the difference in complete wound healing, follow-up duration must be long enough. For example, in a 9-week RCT of silver-containing dressings in the management of infected venous ulcers by Dimakakos et al,^[28]^ statistical differences in complete wound healing were observed. It is suggested that future studies should lengthen the intervening time and increase the frequency of wound assessment in order to obtain higher quality clinical experimental data.

In the absolute reduction of wound area, although only descriptive analysis was performed due to the differing presentations of the outcome, the results of the 2 4-weeks RCTs were in favor of silver dressings. In the study of Lazareth et al,^[13]^ after week 4, all patients in the silver dressing group switched to the non silver-containing contact layer for 4 additional weeks treatment. At week 8, the median absolute wound area reduction was still significant different between the 2 groups (*P* = .002). With regard to relative wound area reduction and wound healing rate, our meta-analysis showed that silver-containing dressings could effectively reduce the wound area (*P* = .03) and accelerate wound healing (*P* = .004). In the study by Senet et al,^[24]^ patients were treated for 6 weeks with either Biatain or Biatain-Ag followed by 4 weeks treatment with Biatain, relative area reduction and healing rate showed significant differences between the experimental group and control group in the subgroup of patients with older and larger ulcers (*P* < .05). And at the 10th week of follow-up, the different of the relative wound area reduction between the 2 groups was more significant compared with the results after 6 weeks treatment. This indicates that the effect of silver appears to continue at least for 4 weeks after the treatment. Similarly, Miller et al pointed out that silver dressings were associated with faster wound healing rates in the first 2 weeks. A systematic review also reported the same evidence^[29]^ and no differences were found on long-term follow-up. These findings suggested that when patients had large leg ulcers or history of recurrent ulcers and rapid reduction in the size of the wounds was desired, silver dressings may be the best choice.

Of note, the results in the experiment conducted by Krasowski et al were quite different from others. This was mainly due to the otinidine dressing used in the trial, which may have stronger antimicrobial activity and lower cytotoxicity compared with silver dressings.^[30]^ Therefore, due to clinical heterogeneity, this study was excluded from the meta-analysis.

As for the infection rate or reduction in infection of the ulcers, descriptive analysis of 4 studies^[13,21,22,24]^ showed that silver dressing had no advantage in controlling wound infection. On the contrary, it is even less effective than the otinidine dressing in the antimicrobial effect, which may be related to the unique antibacterial properties of otinidine dressing.^[22]^ As infection is an important factor in chronic wound healing, it is necessary to carry out more clinical studies to quantify this outcome and explore differences between various antimicrobial dressings in the treatment of chronic wounds in the future.

Previous systematic reviews and meta-analyses have not always supported the role of silver-containing dressings in the management of chronic wounds.^[15,31,32]^ However, consistent with the current results, several studies have proved that silver dressings have great advantages in accelerating wound healing and reducing wound area in certain circumstances;^[16,17,33,34]^ even so, few RCTs have found statistical differences in complete wound healing due to the lack of high-quality long-term follow-up clinical data. Carter et als study,^[16]^ which included not only VLUs, but also other types of leg wounds, showing that silver treatments and silver dressings can significantly reduce the size of the wounds. However, no significant advantages were found in complete wound-healing and healing rates. A recent Cochrane systematic meta-analysis^[19]^ stated that silver dressings may increase the probability of VLU healing, compared with non-adherent dressings. However, when compared with foam dressings and hydrocolloid dressings, it is unclear whether the intervention increased the probability of healing. Different from other studies, this study focuses on the effects of various silver dressings on the wound of VLU compared with all other non-silver dressings. Our results strengthen the proposition that silver containing dressings can improve the healing of chronic wounds, especially the chronic VLU wounds. In addition, silver dressings also have good acceptability and tolerance, and can reduce pain and wound exudates.^[33,35]^ Some studies have pointed out that silver dressings can improve patients health-related quality of life, and are cost-effective in wound treatment,^[17,36,37]^ whereas other studies have reported that there are no differences when compared with other dressings.^[8,38]^ These conflicting conclusions may be due to the fact that wound types and dressings included in each study were different. Therefore, it is necessary to evaluate specific chronic wounds in order to get more accurate results, and to conduct more clinical trials to compare the effects of different silver dressings in wound management in the future.

Though we conducted a comprehensive search of the literature on the treatment of VLU with silver-containing dressings, the current study still had some limitations. First, due to the limited number of high-quality studies retrieved, effective sub-group (different silver dressing group or antibacterial dressing and non-antimicrobial dressing group) analysis could not be performed; hence, we cannot draw conclusions about which silver dressing is the most effective for VLU and whether silver dressings are more beneficial in the management of chronic VLU than other antibacterial dressings. Second, of the 8 studies included, 4 studies were conducted for 4 weeks and 1 for only 3 weeks. Third, although meta-analysis showed that silver-containing dressings could significantly reduce the wound area and accelerate the healing rate of VLU, more RCTs are needed to support this result. In addition, Egger et al^[39]^ have emphasized that if double blindness is not adopted or insufficient distribution concealment exists in the experiment, the results would be overestimated by 15% and 30%, respectively, which means that the therapeutic effect of silver dressings on chronic VLU may have been exaggerated in this evaluation. Nevertheless, our study provides a more accurate basis for patients with venous ulcer to choose silver dressing, and provides a certain direction for future research.

In conclusion, the results of this meta-analysis showed that the function of silver dressing in VLU was similar to that in other chronic wounds. Though no differences were observed in the rate of complete wound healing, which was probably due to the lack of long-term follow-up data, there was sufficient evidence that silver-containing dressings could accelerate the healing rate of chronic VLU and improve healing in a short time. Future research should focus on extending intervention time and enlarging sample size, lay emphasis on differences between various silver dressings and whether silver-containing dressings have unique advantages in chronic wound management when compared with other antibacterial dressings.

## Author contributions

**Conceptualization:** Liping Tan.

**Data curation:** Minyan Zhao.

**Formal analysis:** Minyan Zhao.

**Funding acquisition:** Liping Tan, Hui Huang.

**Investigation:** Minyan Zhao.

**Methodology:** Minyan Zhao.

**Project administration:** Liping Tan.

**Resources:** Dongting Zhang.

**Software:** Dongting Zhang.

**Supervision:** Dongting Zhang.

**Validation:** Liping Tan, Hui Huang.

**Writing – original draft:** Liping Tan, Hui Huang.

**Writing – review & editing:** Hui Huang.
